# “My first 48 hours out”: drug users’ perspectives on challenges and strategies upon release from prison

**DOI:** 10.1186/s12954-021-00480-w

**Published:** 2021-03-12

**Authors:** Daniela Jamin, Wouter Vanderplasschen, Orphée Sys, Marie Jauffret-Roustide, Laurent Michel, Philippe Trouiller, Andreia Neisa, Mariana Homen, Vânia Mendes, Heino Stöver

**Affiliations:** 1 Institute for Addiction Research, Frankfurt University of Applied Sciences, Frankfurt, Germany; 2 Department of Special Needs Education, Ghent University, Ghent, Belgium; 3 Cermes 3 (Inserm U988/CNRS UMR 8211/EHESS/), Université de Paris, Paris, France; 4 Baldy Center for Law and Social Policy, Buffalo University of Social Sciences, New York, USA; 5 CESP, INSERM UMR1018, University Paris - Saclay, Pierre Nicole Centre, French Red Cross, Paris, France; 6 APDES, Agência Piaget Para O Desenvolvimento, Villa Nova de Gaia, Portugal

**Keywords:** Harm reduction, Prison, Overdose, Drug use, Risk behaviour, Release, Recovery

## Abstract

**Background:**

Prisoners report much higher prevalence rates of drug use and more harmful consumption patterns than the general population. People who use drugs have above-average experiences with the criminal justice system in general, and the prison system and subsequent release situations in particular. Release from prison is associated with increased mortality rates among drug users due to the risk of overdose. The EU-funded project ‘My first 48 hours out’ aimed to address the gaps in continuity of care for long-term drug users in prison and upon release, with a special focus on drug user’s perspectives on needs and challenges upon release.

**Methods:**

A multi-country (Belgium, France, Germany and Portugal) qualitative study was set up to explore drug users’ perceptions of drug use and risk behaviour upon prison release, experiences of incarceration and release, and strategies to avoid risks when being released. In total, 104 prisoners and recently released persons with a history of drug use participated in semi-structured interviews and focus groups discussions on these topics.

**Results:**

Respondents pointed out that there are numerous challenges for people who use drugs when released from prison. Lack of stable housing and employment support were frequently mentioned, as well as complex administrative procedures regarding access to services, health insurance and welfare benefits. Besides structural challenges, individual issues may challenge social reintegration like ‘old habits’, mental health problems and disrupted social networks. As a result, (ex-)prisoners adopt individual strategies to cope with the risks and challenges at release.

**Conclusion:**

Measures to prepare prisoners for release often do not focus on the individual and specific challenges of persons who use drugs. Psychosocial and medical support need to be improved and adjusted to drug users’ needs inside and outside prison. To improve the quality and continuity of care around release, the perspectives and coping strategies of people who use drugs should be used to better address their needs and barriers to treatment.

## Background

Prisoners report much higher prevalence rates of drug use and more harmful consumption patterns than the general population [1]. Lifetime prevalence among prisoners in the European Union for using any illicit substance before imprisonment is estimated between 16% (Romania) and 79% (England, Wales and the Netherlands), and between 15% (Finland) and 39% (Spain) for using heroin [2]. Up to 37.8% of all prisoners declared to have injected drugs at some point in their lives; while up to 31% stated to have injected drugs during imprisonment [2]. Although drug use as such is not a criminal offence in most European countries, most regular opioid users report multiple experiences with the prison system. As a result, people who use drugs have above-average contacts with the criminal justice system in general, and the prison system and subsequent release situations in particular [3].

Release from prison is associated with increased mortality rates among drug users due to the high risk of overdose [4–10]. Sixty per cent of all drug-related deaths occur within 12 weeks after release from prison [8] and 20% of drug-related deaths are connected with prison release or leaving treatment [11]. In England and Wales, released female prisoners were 69 times more likely to die of drug-related causes during the first week after release, while released male prisoners were 28 times more likely to decease than people from the general population of the same age and gender [4]. In particular, the immediate period after release is a critical period for support and action, when cooperation between health and social services in prison and the community is the key factor to ensuring continuity of care (throughcare) [12–14]. Targeted interventions can save lives and build pathways towards engagement in further treatment and recovery [15], but coordination and continuity of care need to be improved in many countries [16]. Preparing prisoners for release starts inside prison and needs to be continued after release, without interruption of health care and social support. Anecdotal evidence shows this is not the case in most countries due to a patchy network of services and ad hoc provision of support at the level of single prison establishments, resulting in enormous differences between regions and countries around Europe [17]. For example, measures that focus on overdose prevention at prison release have only been reported in 15 out of 33 countries in the EU [11].

The multi-country research project ‘My first 48 hours out—comprehensive approaches to pre-and post-prison release interventions for drug users in the criminal justice system’ (2017–2019) was funded by the European Commission (CHAFEA Grant Nr: 677085) to address gaps in the continuity of care for long-term drug users in prison and upon release. The project aimed at promoting life-saving interventions for the prevention of overdose, reducing risks related to drug use and establishing recovery pathways that are not interrupted upon release [18]. This paper focuses in particular on the perceived continuity of care as experienced by drug users who are/were imprisoned and on their perspectives of challenges associated with release and strategies applied to initiate/maintain recovery beyond the prison walls.

## Methods

A multi-country (Belgium, France, Germany and Portugal) qualitative study was set up to explore drug users’ perceptions of drug use and risk behaviour upon release from prison, their experiences of incarceration and release, knowledge of risks and overdose prevention, and individual risk reduction mechanisms and strategies they apply when being released. The choice of these four countries was primarily based on previous successful collaboration in a project on access to harm reduction measures in prison and on the diversity in harm reduction and health care policies in prisons in these countries, representing practices from the West, Center and South of Europe [19]. Some notable differences between these countries in the organisation of prison health care for persons who use drugs are mentioned in Table 1. Importantly, the possession (for personal use) of all drugs was decriminalised in Portugal in 2001, which may have led to slight differences in the profile of (formerly) incarcerated Portuguese drug users as compared with those in the other countries.

**Table 1:** Differences between the countries regarding health care and drug treatment before/during/after prison

Topic	Germany	France	Belgium	Portugal
Cost free access to OST? (in community)	Yes	Yes	No	Yes
Access to Naloxone? (in community)	Yes (limited)	Yes	No	No
Responsibility for care organization? (inside prison)	Ministry of Justice	Ministry of Justice	Ministry of Justice	Ministry of Health
OST available in prison? (inside prison)	Not in all	Yes	Yes	Yes
Take home naloxone available? (inside prison)	No	Yes	No	No
Certificate providing access to health insurance? (at release)	No	Yes	Yes	Not needed

### Sample

The study sample consisted of 104 respondents (Table 2). Individual interviews (*n* = 84) were administered among 43 prisoners and 41 former prisoners, including 12 women and 72 men. In addition, 5 focus groups were organised in prisons, with in total 20 participants (5 women and 15 men). Female prisoners appeared to be more difficult to reach because they are underrepresented in the prison system compared to males and not all prisons have facilities for women. Female respondents were only recruited in France and Germany. The average age of participants was 36.7 years (range 19 to 54 years). Participating prisoners and ex-prisoners had served on average 5.3 detention periods (range 1–35) and spent—on average—a total of 86.4 months in prison (range 1–336 months). Former prisoners had been released from prison for an average of 2.2 months at the time of the interview. The primary drugs used by most respondents were cocaine and heroin, often in combination with other substances like crack, amphetamines, ecstasy and/or cannabis.

**Table 2:**
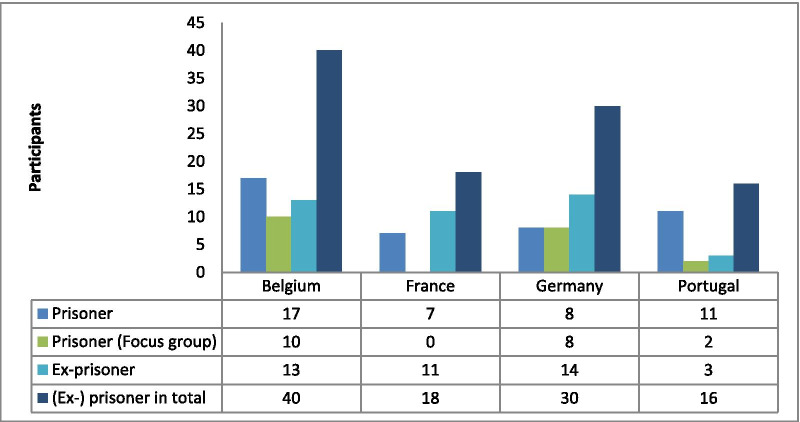
Number of prisoners and ex-prisoners participating in each country in interviews and focus groups

### Data collection

The research took place between May 2017 and August 2018 in six prisons in the four participating countries (two in Germany, two in Belgium, one in France and one in Portugal) and in several services that support people who use drugs (in prison), e.g. inpatient and outpatient drug treatment centres, low-threshold services, opioid substitution programs in the community, prison health and social services.

Prisoners were recruited through prison staff after the researchers received authorisation for the interviews in prisons. While the researchers in Belgium had direct access to prisoners to inform them about the study, the researchers in France, Portugal and Germany had to rely on professionals inside prison to approach eligible interviewees. In order to be eligible for the study, prisoners had to meet the following criteria: (1) being a recent and/or regular user of illicit drugs (other than cannabis), (2) having served at least one prior prison sentence, (3) master the country language sufficiently to do an interview, and (4) be willing and available to participate in an interview. Recruitment strategies differed between the four countries according to the specific context and policy, but the eligibility criteria and methodological approach were the same, in order to allow a comparative analysis.

In all cases, eligible participants were informed in various ways about the project (e.g. through personal contacts, flyers) and participation was completely voluntary. In case individuals were interested, they signed an informed consent form and the interview could take place immediately or at a fixed moment in a dedicated place without the presence of staff, video or any other control measure.

Former prisoners were recruited through treatment and harm reduction services in the community. These organisations were approached via email, telephone and personal contacts to help with the recruitment of recently released prisoners (up to five months maximum). In some cases, flyers were distributed in these organisations, so that ex-prisoners could contact the researcher or the organisation’s social workers if they wished to participate in the study. Former prisoners were eligible if they (1) had served at least one prison sentence (the last one maximum of five months ago), (2) were recent and/or regular users of illegal drugs (other than cannabis), (3) spoke enough Dutch, German, French or Portuguese to participate in the interview (according to the primary language in each of the participating countries), and (4) were willing and available to participate in the study.

All interviewees participated voluntarily based on an informed consent and could stop the interview at any time. In all cases, the informed consent form was explained before the start of the interview. Potential participants were informed that there was no obligation to answer all questions. If the person agreed with the content of the informed consent, he/she could sign the form and the interview could start. Nearly all interviews were recorded using an audio recorder. Afterwards, the interviews were transcribed and anonymised. Only in Portugal some interviews were not recorded, since there was no authorisation to do so in prison. In these specific cases, the interviews were directly documented by the interviewer. Following the end of the interview or focus group discussion, participants received a small incentive (10 EUR) in the form of cash, a gift voucher or tobacco for participating in the study.

Prisoners and former prisoners were interviewed using a semi-structured interview. In addition to the interviews, focus groups were organised in three countries to make authentic utterances more likely during the shared interaction and to allow the course of the discussion to point to topics that are important to the group. The following topics were discussed: drug use and risk behavior in prison and upon release, knowledge of the risks of overdose and methods to deal with overdose, individual strategies to deal with drug use, and related risks and experiences with release. Both methods can be regarded complementary and the interviews were used to assess individuals’ perspectives and to obtain information about individual strategies and statements, while the focus group discussion were organized to stimulate discussion around these themes and to better contextualize the study findings. The topic lists used for the semi-structured interviews and focus groups were translated from English into the country language and were identical in the four countries.

### Data-analysis

Based on a first content analysis of the interviews and focus groups, a common tree structure was developed for structuring the data analysis in all four countries. The themes and subthemes were selected in close collaboration between the researchers from all four countries. Data-analysis was performed using the qualitative software program NVivo, by assigning meaningful text segments (nodes) to the tree structure. Perspectives and experiences of (ex-)prisoners that were collected during interviews and focus groups were analysed thematically in each country and then merged into one common analytic framework.

Country-specific results are discussed in separate country reports and were not considered for this paper, as the researchers in the four countries found very similar results and identified the same core themes during the analysis. In order to illustrate the results, we use literal quotations from the semi-structured interviews: quotations from prisoners are marked with the abbreviation PR, while quotations by former prisoners are marked as EPR. In addition, M/F indicates whether a man or woman mentioned this quotation. The country of the respondent was abbreviated as BE for Belgium, FR for France, DE for Germany and PT for Portugal. To preserve respondents’ anonymity, we decided not to report participants’ age.

## Results

### Challenges upon release

Respondents make clear that it is very difficult to return to the rush of present-day society and to get ‘up to date’ with the latest developments, especially during the first days and weeks after release. The longer one has served a sentence, the more difficult this is. It feels like an enormous confrontation with the speed and time pressure in society, which is a huge contrast with the ‘structure and order’ in prison, where nothing seems to change. According to the respondents, handling the first days outside is very hard and some have the feeling that they have to learn again how to live and to organise their daily new routine, especially how to behave in interaction with others in society.

“(…) At the same time it is confusing. We are closed in here for so long that it seems that we no longer belong to this world” (PR, M, PT)

A lot of things are expected immediately after release, like administrative settlings, making contacts with people in society and managing a life outside prison. In this regard, some respondents indicate a lack of internal motivation to contact services and engage in activities, or that they are struggling to accept support.

One of the main coping strategies upon release is to cling to old habits (‘automatic reflexes’), in order to deal with the transition, which often meant returning to their previous activities and environments, such as drug use, friends who use drugs, or criminal involvement. Respondents state that returning to former social networks, when they mainly consist of persons involved in drug trafficking and dealing, is mostly a result of a difficult period after/upon release.

“According to me, the big challenge is to reconnect with people. Again, it depends. If the person has done three months, that’s fine. But for people who have done more than a year, (…), it is not easy to return […].The stress, the cars driving, all the noise,.., all that stuff is kind of stressful. The person may be inclined to consume just to calm down.” (EPR, M, FR).

The family and social network can also impact the release experience in a negative way. Negative emotional experiences towards family or friends who do not want to meet them after release, fear of stigmatisation by their social networks and a lack of social support are reported as negative experiences upon release. The absence of close relatives or their reactions may lead to strong disillusion.

“Upon the last release, I had to see my family who was supposed to pick me up. They didn’t pick me up, and it didn’t go well. I had emotional expectations, I thought I’d see them, they didn’t come. (…) At this moment, I was out of my mind. So what did I do? I started using again. I didn’t go to my treatment centre, so I was on the run and I eventually came back here [in prison].” (PR, M, FR).

The interviews show that the main fear of prisoners is a relapse after release, because some respondents anticipate that they will not have the power to address the difficulties and challenges upon release successfully.

In addition to challenges at individual level, structural bottlenecks may further complicate individuals’ reintegration after release from prison. Housing and employment are major challenges for most respondents. Having sources of support in the community (like drug treatment services, friends and family) is seen as very helpful in terms of financial/logistic support, as well as for providing shelter and social and emotional support. Arranging paperwork is another major challenge in the first days after release. Obtaining a health insurance and access to OST appear to be particularly difficult. Respondents also mention the mental harm after a prison sentence, and sometimes the wish to go back to prison. The lack of coordination and attunement between medical and psychosocial support inside and outside prison is reported in all countries.

Despite the importance of adequate housing, some respondents indicated that they lost their flat during detention and did not know where to go after release. Often participants had no proper housing solution, even though housing was one of their main concerns. Some struggled to find emergency accommodation in a shelter and others were forced to sleep rough. Others managed to arrange accommodation in a low-threshold service or in a treatment centre specifically designed for ex-prisoners with a history of drug use (like in France). A few participants obtained private accommodation, which gave them a sort of confidence and protection.

“I want to treat myself, I want to feel good so I can go on with my life properly. What I need is to feel it, to have a comfort zone. In prison, I have a comfort zone, I don’t need to worry. Being at home with my parents, I might not need to worry, I would feel useful and gradually, I think I would take my life back.” (PR, M, FR).

Another major challenge upon release is finding a job and/or training/education. Respondents indicate that it is very hard to find employment outside prison when you have a criminal record. Employment is often regarded an important aspect of reintegration because it provides a daily structure, allows to perform a task in the community and provides financial resources.

“After imprisonment, the biggest difficulty I encounter is the financial situation and employment.” (PR, M, PT)

The complexity of administrative procedures is also frequently criticized by interviewees. To obtain basic requirements and support such as identity documents, health insurance and welfare benefits, it is necessary to have a stable life routine and a good understanding of the administrative system. Respondents often had to start these procedures from zero: without a fixed address, bank account or proof of ID. Therefore, this often laborious task is regarded a seemingly impossible, which brings about disappointment or frustration.

Various respondents described the huge gap between the support they received inside prison and the support they got once out of prison. They experienced a difficult transition from relatively accessible, regular and well-defined support inside prison to a more volatile and sporadic support in the community. It appears as if care inside prison was somehow ‘passively’ received, while health care outside prison requires much more motivation, persistence and action. A treatment gap regarding opioid substitution treatment illustrates this problem: in prison, users are called into the medical unit for their medication each day, and a strict routine is installed, but once they are released they need to find a way to obtain OST without health insurance, and sometimes without prescription. In some countries, health services in prison provide medication for two or three days on the day of release, but respondents didn’t find this was enough to make the bridge between prison support and community treatment.

“The problem is that I only had for two days of methadone on me and since I had to go to X [an addiction treatment centre]at the third day, not having treatment anymore […] could be complicated. So, the evening before, I took half of the medication and saved the other half for the morning. But it’s true that in the evening, I wasn’t very well and I went back to my neighbourhood. I smoked a little heroin to remove the craving. It wasn’t really a desire I would have had if I had had all my treatment, but that’s the way it went down.” (EPR, M, FR).

A frequently heard negative story at release is that prisoners are released unexpectedly, especially in case of a short prison sentence. Since the release date is not known in advance, prisoners often end up on the street all of a sudden. Respondents in all countries also reported that they got no support at this point and were ‘kicked out’ of prison only with a bag and no plan to go. Some indicate that they would rather have stayed inside than to be released without a plan.

The majority of respondents indicate that preparations for release are often minimal. Most (ex-) prisoners felt uncomfortable on the day of release due to the uncertainty about not knowing what situation they would face afterwards. Some state that they lost their flat during imprisonment or found their flat in bad condition after release. In some cases, respondents reported that they were released on a Friday and that they had to wait until the next weekday for continuing their OST. Most respondents indicated that they went or would go to shelter homes or low-threshold drug treatment centres upon release, which are associated with drug use, dirt and unhelpful contacts. However, they mostly found a place to sleep at these places and reported useful contacts with social workers and the possibility of accessing OST.

Respondents indicate that if a prisoner serves a short sentence or chooses to serve the whole sentence (without being released early through provisional or conditional release), usually no reintegration plan is made up with the prisoner. In case of a longer sentence, prisoners are entitled to parole and exit permissions before release, which facilitates making arrangements. They can work on a rehabilitation plan: searching for a house, doing paperwork, looking for a job and restoring ties with the family are things that can already be picked up before release.

### Reasons to use drugs at release

Most participants recall having used drugs in the two weeks following their last release. Using drugs at release can be considered as a strategy to cope with the uncertainty and stress caused by the fact that their release was not well prepared.

Respondents point out the difference between the mindset they had before release and the one they had after consuming drugs again: even when they are convinced they will stick to the treatment and not use again, they gradually make one concession after the other and they very quickly find themselves in the same situation as before their incarceration. Some ‘experienced’ respondents mention an evolution between the first releases from prison and subsequent ones: when they were younger, they felt the need to ‘party’ for two or three days after release to compensate as quickly as possible for the deprivation they endured during detention. Later, they tend to be more careful about it and resist this urge because the switch to regular use could happen very quickly. (Ex-)Prisoners indicate that boredom and being without housing are important pitfalls to start using again or to continue using after release.

“First of all, a roof above your head, because for 99.9 per cent there is a chance that… Who wants to live on the street? As a drug addict you will relapse, because they cannot cope with that and [will use] for feeling better, even though it’s the wrong thing to do […], feeling stronger at the moment.” (EPR, F, DE).

The importance of a daily schedule and having something to do (a job, hobbies) is often quoted by participants. A pitfall for relapse is having contact with the (old) user network. Often, there is no other (drug-free) network on which they can rely the days following release. Respondents’ networks often include people who use drugs and are thus seen as acting as a stimulus to use again. Persons who returned to their old neighbourhood to seek social contact and support feared that they would run into friends who are still using drugs. Shelters providing emergency accommodation also increase the chance of meeting people who use drugs and are considered by the participants as a risk factor for relapsing into drug use. Also, when one is lonely and without (social) support at that moment, the step towards drug use is easily made.

“I was released and I wanted to pick up again the outpatient drug treatment I had before detention, but my therapist was on leave. So, I wanted to work on it for three weeks, but I couldn’t and then I lost control. I had a relapse. I met an old friend who was still using speed and […]. Last time, I was free again and I did everything well: I requested financial support and got two weeks of support. But the third week, they told me I had to find my own way. I went back to my brother and used drugs, I shouldn’t have done that. But it is difficult if you are in that circuit. Certainly, if your brother is a user, most of my family are users … Where can I go? You’re in the middle of a struggle… You come out, have no home, I could stay with my brother, but I was also alone there, so what do you do to be able to talk to someone? I went to my cousin, but he also used drugs there. I lost myself. I thought I could handle it, but first it is one line [of coke], another line half an hour later and like this you’re back again on drugs.” (PR, M, BE).

Participants pointed out that lack of activities immediately following release is an additional risk factor for drug use. Even when health support or administrative procedures were initiated, they report long periods of waiting for social or medical support to be able to move forward. During this post-release period, they often lack a sustainable housing solution and cannot seek employment as their administrative situation is not regulated yet. Given this uncertain situation and difficult transition, having nothing to do contributes to anxiety and/or disorientation.

Some interviewees indicate that they had a very strong motivation inside prison, but felt substantial craving after release. Also being ‘clean’ (especially after no access to OST inside (e.g. in Germany)) can be an important pitfall for using drugs outside, as a number of respondents stressed the ‘need’ for drugs or a ‘good cocktail’ (combination of cocaine and heroin) after release. In some cases, increased consumption is reported after release, others use less than before imprisonment.

“And these thoughts you had what to do after release: work, new life, looking for a flat and this and that. All this just disappears on the day of release. You’ll forget that soon after you leave the prison a few yards away. That’s so bad. That is madness. And then, again and again drugs.” (EPR, M, DE).

“But sometimes, when you didn’t use drugs for a long time, there is also craving. Then you have to satisfy the addiction. Yes, and that’s a real force to do this sometimes.” (PR, M, DE).

Difficulties that (ex-) prisoners encounter when arranging things like employment and housing are not helpful either and may trigger them to use again. Moreover, a number of respondents indicates that if medical treatment is not continued after release (because of lack of health insurance), relapse is very likely.

“If you have a lot of money at release and no doctor anymore [for prescribing OST], so what do you do first? You think: “Yes, OK. I’m just getting the bare necessities, so I’m not on withdrawal.” And what do I do then? That does not last that long.” (EPR, M, DE).

### Individual strategies to cope with risks associated with drug use

The interviews showed that respondents apply specific, individual strategies to cope with risks associated with drug use. These coping strategies exist at individual level, but some strategies are mentioned by several respondents.

Some (ex-) prisoners indicate that it was helpful to change their lives. Changes in social life (daily structure, work, leisure time), contacts (friends, dealers) and housing were reported as being particularly helpful for changing drug use patterns. This strategy is also applied and experienced as helpful in the community: (ex-) prisoners break up contacts with drug users and stay away from their old neighbourhoods. Having (new) clean contacts is definitely thought to be helpful. Some also report a need to know more about their personal risk factors for drug use, while some indicated that a realistic perspective is helpful (with abstinence not being possible for them).

“Hand on heart, I cannot do it without a substitute. What’s so reprehensible to say is: I cannot do it. That’s it, and I live with it legally and I can live with it. I can also build a life for me with a substitute. And that’s what I recognised and not thought: what do others think? I do not care what others think. That’s my life.” (PR, F, DE).

Some (ex-) prisoners state that they intentionally avoid visits from some people in prison (e.g. friends) to protect themselves from having drugs being brought in. Another strategy to stay away from drugs in prison is having something to do. Participating in organised activities such as fitness, sports or cooking helps prisoners to relax, reduce stress and distract their thoughts. According to several respondents, having a structure (with daily tasks and activities) is also very helpful in the outside world.

“I want to change my life for myself, so I stay away from the people who are still using drugs here.” (PR, F, DE)

According to several respondents, having a person of trust and/or children is of great help outside prison. Carefully preparing for someone’s release is seen as a necessary condition. Several prisoners indicate that someone is lost when arrangements are only made upon release. Moreover, (ex-) prisoners state that it is important to find a way to cope with the prison period and to recover from the stress they experienced in prison.

“They [fears of overdose after release] really exist and I thought so too, because I’m scared, but the only thing that helps me is that I do not consume when I go to my children. I know that I will not consume, because when my children are there, their presence always makes me forget everything else.” (PR, F, DE).

In specific cases, (ex-)prisoners apply strategies like setting small goals for themselves, regular abstinence periods, or organising finances and administrative tasks before taking drugs after release.

Individual harm reduction strategies are named regarding overdose prevention. Respondents indicate that it is common sense not to use too much or to take overly large dosages. A proven strategy is to stick to a certain dose. Another respondent states that you should only use one third of the normal dose, if you haven’t used for a long time. It is recommended to build up consumption gradually, first by using a little bit and then a little more later, to reduce the risk of overdose. Another strategy is to use a different mode of administering drugs immediately after release, such as smoking instead of injecting.

“To use the drug in moderation. If I want to eat everything at once, I'm bound to go down. After using, wait a bit.” (PR, M, PT)

“If I use drugs, I do me half (of the usual dosage) or less than half and then I wait. What happens when I realise, oh, there is something wrong? Then I stop.” (EPR, M, DE)

Several (ex-)prisoners mention that is important to ‘know the drugs that you are using’. A proven strategy reported in Belgium [only available outside prison] is to have the drugs tested first to know about purity and quality. An overdose can also be prevented by never using alone or using in a consumption room, where someone else can help you if necessary. In Portugal, respondents specifically argue for the provision of safe drug consumption sites as a structural measure to prevent overdose.

“Knowing what you are using. Many years ago, I have seen people injecting and dying. They do not know, they think they are injecting cocaine and it is not cocaine. They do not really know what they are using.” (PR, M, PT).

“Take less. … and always with another person, that one is there. […] That’s the first, if you go away (use) alone and you do not know the stuff.” (EPR, M, DE)

Some respondents mentioned emergency measures in case of an overdose like using salt water or naloxone.

“I've seen a person with such a problem. The boy who was injecting stood there and never got up again. The other who was smoking took salt water and stuck it in and he woke up. I also know that naloxone prevents it.” (PR, M, PT).

Finally, abstinence was also reported by some participants as the best way to prevent an overdose.

### Positive and helpful measures at release

Most respondents had more negative than positive feelings regarding release, except Portuguese respondents. Having a home and good contacts with family and friends are obvious positive factors associated with release. Having ‘someone who waits’ in the community helped them to ‘keep their mind off drugs’ and was reported as being very helpful and supportive in the period immediately following release. Respondents note that a supportive social network is the main factor in enabling positive reintegration, since they are the ones caring for their needs at release, namely social support, housing, supplies and finances. However, the majority of the respondents from this sample did not have such a prosocial network.

“I came to the conclusion that family is very important for any type of rehabilitation or reintegration, because they support such basic things as food or housing.” (PR, M, PT)

Some respondents associate release with a sense of freedom.

“The moment of departure is a unique moment, so much joy that no matter how angry you are you forget everything. I missed things when I was in prison. When you walk out the door, you have access to family, the most beloved ones. (…) The door being open is all good.” (PR, M, PT).

At structural level, respondents reported diverse experiences. The majority of respondents mentioned bad experiences, but some also had good ones. Besides housing, the major protective factor appears to be finding a job after release. Work is associated with strong and multiple support, since it is linked with reinsertion, an income, social contact, and, most of all, an occupation. One of the ex-prisoners even presents work as a way to ‘find exhaustion at night’, and to get back to a normal ‘rhythm’ in his life.

Regarding access to health care, some participants convey positive experiences of coordination of care between professionals outside and inside prison, allowing them to feel more secure just before and after release. Some mentioned the leave they obtained from the prison administration to prepare their release and to visit a residential treatment centre for a day. A methadone prescription provided before release by the prison medical service and/or treatment centres providing free OST or OST for persons who pay themselves were mentioned as highly practical and reassuring by the interviewees, as receiving OST is one of the major challenges upon release if individuals have no health insurance.

Some respondents refer to specific education and trainings inside as a positive element for reintegration, including receiving information and identifying interests for leisure activities in the community. Besides training, some respondents mention that residential therapeutic programs have a positive effect after release, in terms of the opportunity to meet other people who recovered and are leading their own life autonomously.

“(…) There, I worked with medical therapists, did small community work and then would move to live in an autonomous reinsertion home. I felt it was important, I met people who recovered and went there at weekends to talk about what they had already achieved.” (PR, M, PT).

Some (ex-) prisoners mention the positive impact of preparation for release. This is often related to following therapy after release or specific support like a social worker outside, case or transition management or the support of family and friends. Only a few respondents report that they experienced an open prison regime, but found it very helpful for learning to live outside.

## Discussion

This multi-country, qualitative study among 104 prisoners and ex-prisoners who use drugs demonstrates that respondents are facing similar specific challenges at the point of release from prison in Belgium, France, Germany and Portugal. As a consequence of these substantial needs, they start to adopt strategies to cope with these individual and structural challenges, in particular when their release is not well prepared by prison and community services. Continuity of support [12], linkage to community services [20] and specific prevention measures [14] (e.g. provision of take-home naloxone kits) are crucial elements for reducing the number of drug-related deaths and promoting social reintegration after release. The conclusions and recommendations drawn from this study fill an important gap, as they are based on the rich experiences of long-term drug users. Although study participants may be regarded quite old and using ‘traditional’ drugs (i.e. heroin and cocaine) compared to newer generations of persons who use drugs, all respondents had experienced multiple detention periods and subsequent releases. They can be considered ‘system survivors’ as they have experienced specific challenges and learnt to cope with these difficulties on their own, in the absence of sufficient support. Consequently, their experiences and perspectives are an important source of information to develop more appropriate harm reduction and social reintegration strategies [21].

Individual (e.g. stress, unrealistic expectations, social network support) and structural (e.g. housing, employment, administrative burden) challenges and barriers affect drug use in prison and after release substantially. Throughcare, a UK approach to improve continuity of medical and psychosocial care before, during and after imprisonment, reduces the risks of relapse and overdose at release [22]. The provision of throughcare and other models of individual case management is important to reduce drug use [23] and to increase the likelihood of re-integration after release [12, 24], especially for prisoners with multiple health and psychosocial needs. The findings from this study clearly show the complex and multiple needs of drug users in prisons across various EU countries, in particular at the point of release, but measures and services to prepare their reintegration are scarce and mostly limited to prisoners who serve long-term sentences. Drug treatment and medical care are important helping resources for persons who use drugs, besides addressing complex psychosocial needs like housing, social support and a day structure [3, 10, 25].

Respondents perceived the continuity of opioid substitution treatment (OST) as having a positive effect on the reduction of drug use and risks related to drug use in prison and upon release. On the other hand, (ex-)prisoners identified that discontinuity of OST or receiving lower dosages as a risk factor for drug use in prison or directly after release. Available studies and guidelines show that medically assisted treatment with OST can reduce the risks of relapse and overdose at release and should be a core element of health care services for prisoners who use opioids [5, 10, 26–28]. All countries that participated in this study provide OST in prison, although dosage and coverage rates differ between countries but also between regions and prisons within these countries [19].

Since several respondents mentioned to have experienced an overdose, the provision of naloxone is—besides providing OST—an important measure to prevent drug-related deaths due to overdose [5]. Naloxone is an opioid antagonist capable of reversing an opioid overdose due to opioids, such as heroin or prescription opioid drugs. Naloxone has been available on prescription for at-risk drug users and their family/friends since 1999 through selected programmes around the world [29]. Some countries have adopted Naloxone as a core strategy in reducing overdose deaths like Scotland, where naloxone is an integral part of overdose prevention at release [30, 31]. A recent study clearly shows that providing drug users with take-home naloxone at release can reduce the number of overdose deaths after release [32]. Based on this study, it appears that only prisons in France provide naloxone kits and training for overdose prevention before release. Prisoners in the other participating countries had no legal access to naloxone. Despite promising experiences reported in Scotland and other parts of the world [31], this strategy of mortality prophylaxis appears to be widely neglected. As the findings of this study indicated, most persons who inject drugs know about the risks of overdose but also need more information to manage overdose situations [33].

The perspectives of the respondents that were interviewed in Belgium, France, Germany and Portugal clearly showed that psychosocial support is regarded as a crucial element to prevent drug use and relapse upon release. While medical care can be provided to all drug users in a rather standardized way, psychosocial support needs to be tailor-made taking into account individuals’ specific needs or situations. Preparation for release (including family, financial, housing and employment issues) as part of a throughcare or continuing care approach can be a key factor to successful reintegration [10, 22]. Challenges at release appear to be rather complex [34, 35], including more individual stressors and triggers and structural elements that may hamper successful reintegration and recovery. On a structural level, respondents emphasized the importance of decent housing, which is often not available and urges ex-prisoners to look for shelter in low threshold centres or with friends where they may be challenged to use again. Finding a job or training and regulating administrative issues are other important challenges for ex-prisoners, which were identified in all countries. On a more individual level, a lack of daily routine, stress and lack of prosocial (non-drug using) social networks were mentioned as critical elements at release. Protective factors that were named by respondents included starting with or continuing treatment after release, the availability of special resources in the community such as a case manager or social worker outside or support from family or friends [5]. Successful linkage to community resources (e.g. therapeutic programs or training centres) is considered crucial for attending these services after release [20, 24]. Moreover, the reduction of boredom, stress and loneliness is anticipated to have a positive effect on drug use. Also, respondents state that providing meaningful activities like offering a daily routine, occupation or work or the organisation of workshops helps to do so. Ultimately, the findings of this study demonstrate that individual, tailor-made preparation for release is crucial for prisoners who use drugs to cope with the challenges which may lead to relapse and overdose at release. Most (ex-) prisoners the sample had experienced more than one prison release. This is shown in their ideas of their own individual challenges and in their individual strategies to cope with the risks at release, which they developed. Individual strategies focus on risk behaviour regarding drug use and overdose as well on challenges upon release. Even though they pointed out that social support (social worker, family, friends) is the most important strategy.

## Policy implications

In most of the prisons that were studied, generic measures to prepare prisoners for release do exist, although these do not take into account the specific and individual challenges of people who use drugs and are primarily targeted at prisoners serving long sentences. Medical and psychosocial support needs to be improved and adjusted to their needs inside and outside prison [19]. To implement ameliorated harm reduction and social reintegration measures, it is important to learn from the individual needs and strategies of people who use drugs as apparent from this study and to clearly illustrate specific needs as identified across the participating countries.

Drug use, related risk behaviour and overdose risks needs to be discussed with prisoners who use drugs/with a history of drug use before release. Prevention programmes, especially inside prison, need to improve knowledge and support among prisoners to cope with factors that lead to drug use in prison or relapse/overdose upon release [14]. The experience of ‘experts by experience’ and peers who use drugs should also be used in prison settings to develop and implement more effective prevention and harm reduction strategies. In order to do so, a range of services and initiatives can be introduced:

- Realistic information, education and communication (IEC) strategies about the risks of relapse after release (in particular peer-based interventions)
- Improved connections between health and social services provided inside as well as outside prison
- Continuity of medication-assisted treatment (e.g. OST) for opioid-dependent prisoners
- (Re-)uptake of medication-assisted treatment for opioid-dependent prisoners before release (approx. six months) to reduce the risk of relapse
- Training on overdose management and provision of naloxone kits before release [5, 30].

In addition, measures at individual level may help to prevent relapse upon release. The availability of and support by a prosocial social network is definitely a protective factor that needs to be prepared for prior to release [5]. Also, measures and regulations that allow prisoners to prepare their release (e.g. by visiting a community treatment program, looking for housing) contribute to their social reintegration. Professional and informal support initiatives should in particular focus on the pre-release period and the days/weeks immediately following release, but it is recommended to extend re-entry support services up to 12 months post-release [5].

At structural level, more efforts are needed to simplify administrative procedures regarding reintegration (e.g. for arranging welfare or unemployment benefits, housing, continuing OST without disruption). Health care reintegration appeared to be particularly challenging in Germany and France, while the Portuguese system of providing free access to usual treatment without delay can be inspiring for other countries. Other measures that may promote reintegration are the provision and continuity of psychosocial support [12], transitional housing or supported housing initiatives for drug user after release, specific drug treatment initiatives addressing the challenges at release from prison and better cooperation between prison-based and community-based social and health services. Ideally, persons who use drugs should be diverted as much as possible from the criminal justice system [5].

## Limitations of the study

Although we were able to recruit a sample of over 100 prisoners and ex-prisoners for this multi-method study, all countries faced several difficulties in engaging respondents due to the slow and hierarchical organisation of prison systems. In Germany, researchers experienced problems in getting access to prisons due to the involvement of different ministries of justice, while in France the research team did not receive authorisation for doing focus groups with prisoners. Despite providing a financial compensation for study participation, it was not evident to recruit many prisoners who wanted to talk openly about their drug use and release experiences. It appeared particularly difficult to engage ex-prisoners in some countries, as they make use of a variety of services and sometimes do not want to be reminded of their prison stay (or do not reveal their status to support workers out of fear for stigma and discrimination). Despite these challenges, we managed to conduct at least 15 semi-structured interviews per country with (ex-)prisoners who recently used drugs and had previous release experiences.

One of the main limitations of the study is that the sample is not very diverse. For practical reasons, we only included Dutch-, German-; French- and Portuguese-speaking participants, while the prison population is much more diverse in most countries. Also, we limited recruitment to a number of prisons per country (two in Germany and Belgium, one in France and Portugal). Also, only a few community services participated in each country for recruiting ex-prisoners. Contact persons in prisons and community services helped to select respondents, except in Belgium and Portugal, where the researchers could approach potential respondents directly. It cannot be ruled out that there was some degree of selection by these gatekeepers. Female prisoners could only be included in the German branch of the study. Finally, the sample of ex-prisoners may be biased by the fact that only individuals who were in contact with some type of community services were recruited for this study. It is possible that released prisoners with a drug use history who were not involved in any of these services or who were involved in different types of services may have other experiences regarding release.

## Data Availability

The datasets generated and/or analysed during the current study are not publicly available in order to protect participant confidentiality, but are available from the corresponding author upon reasonable request.
